# The Lipid Profile and Mortality Risk in Elderly Type 2 Diabetic Patients: A Ten-Year Follow-Up Study (ZODIAC-13)

**DOI:** 10.1371/journal.pone.0008464

**Published:** 2009-12-24

**Authors:** Kornelis J. J. van Hateren, Gijs W. D. Landman, Nanne Kleefstra, Susan J. J. Logtenberg, Klaas H. Groenier, Adriaan M. Kamper, Sebastiaan T. Houweling, Henk J. G. Bilo

**Affiliations:** 1 Diabetes Centre, Isala Clinics, Zwolle, The Netherlands; 2 Langerhans Medical Research Group, Zwolle, The Netherlands; 3 Department of Internal Medicine, Isala Clinics, Zwolle, The Netherlands; 4 Department of General Practice, University Medical Center Groningen, Groningen, The Netherlands; 5 General Practice Sleeuwijk, Sleeuwijk, The Netherlands; 6 Department of Internal Medicine, University Medical Center Groningen, Groningen, The Netherlands; University of Tor Vergata, Italy

## Abstract

**Background:**

The precise relationship between the lipid profile and mortality in elderly patients with type 2 diabetes mellitus (T2DM) remains unclear. The aim of this study was to investigate the relationship between the lipid profile over time, and mortality in elderly patients with T2DM.

**Methods and Findings:**

In 1998, 881 primary care patients with T2DM aged 60 years and older participated in the ZODIAC study, a prospective observational study. The cohort was divided into two age categories: 60–75 years and older than 75 years. Updated means of all lipid profile indices were calculated after a median follow-up time of 9.8 years. These values were used as time dependent covariates in a Cox proportional hazard model. The cholesterol-HDL ratio and LDL-cholesterol were positively related to both all-cause and cardiovascular mortality in the low age group. In contrast, except for the triglyceride level, none of the other lipid profile indices were related to all-cause mortality in patients aged over 75 years. The mortality risk decreased by 17% (95%CI: 5% to 27%) for each 1 mmol/L higher serum level of triglycerides. The relationships between the various lipid profile indices and cardiovascular mortality were not significant. However, the results were different after stratification for diabetes duration. In the subgroup of elderly patients with a diabetes duration of 8 years and longer, higher lipids were predictive of increased cardiovascular mortality. The main limitation of this study is its observational design, which prevents us drawing conclusions about causality.

**Conclusion:**

Although the lipid profile was not predictive in the overall group of elderly patients, higher lipids were related to increased cardiovascular mortality in patients with diabetes of long duration. In order to make valid recommendations concerning lipid-lowering treatment, a randomized controlled trial or a meta-analysis concerning this specific population is mandatory.

## Introduction

Type 2 diabetes mellitus (T2DM) and dyslipidemia are important risk factors for cardiovascular disease [1], [2]. Randomized controlled trials have clearly demonstrated the positive effects of lipid-lowering treatment (LLT) in T2DM, as shown in two recent meta-analyses [3], [4]. Therefore, treatment with lipid-lowering drugs is recommended for virtually all patients with T2DM in the various guidelines [5]–[7]. Although part of these studies included patients older than 75 years, no separate analyses were performed for this age group. The only randomized controlled trial specifically designed for the elderly (70–82 years) showed a reduction in cardiovascular disease risk [8]. This reduction was largely attributable to positive effects in the secondary prevention group, as shown in a post-hoc analysis. The risk of cardiovascular disease was not reduced in patients with a history of diabetes, although the number of patients with diabetes was probably too small to permit accurate interpretation of the treatment effect [8]. Based on the current evidence, the question therefore remains whether there is a benefit of lowering lipids in the elderly diabetic population. This especially holds true for primary prevention. Also, in a cohort of patients form the general population without verified coronary heart disease, higher cholesterol levels were not related to an increased risk of coronary heart disease [9]. Some other longitudinal studies, including the Framingham study, have even observed increased all-cause mortality rates with lower total cholesterol levels in the general elderly population, regardless of the diabetes status [10]–[14]. We aimed to explore the association between the lipid profile and the risk of mortality in a prospectively designed cohort study of elderly T2DM patients.

## Methods

**Figure 1:**
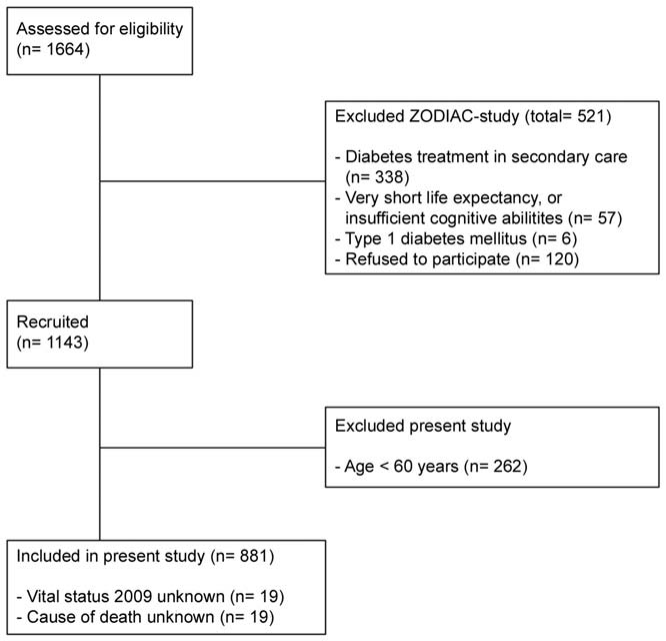
Flow diagram showing the selection process of the ZODIAC and the present study.

This study is part of the ZODIAC (Zwolle Outpatient Diabetes project Integrating Available Care) study; the design and details of which have been presented elsewhere [15]. In this study, general practitioners are assisted by hospital-based nurses specialized in diabetes in their care of patients with T2DM. In the first year (1998) of the study, 1664 patients were assessed for eligibility, and a total of 1143 patients agreed to participate in the study (see flow diagram in figure 1). For the present study, we selected all patients aged 60 years and older (n = 881). Baseline data consisted of a full medical history including macrovascular complications, medication use, and tobacco consumption. Laboratory and physical assessment data, such as lipid profile, serum creatinine levels, the urinary albumin-to-creatinine ratio, blood pressure, weight, and height were collected annually. Total cholesterol, HDL-cholesterol as well as the triglyceride level were determined using standard hospital procedures. LDL-cholesterol was calculated using the Friedewald equation [16]. Patients were not specifically instructed to collect a fasting lipid profile at that time. Early 2009, the life status and cause of death were retrieved from records maintained by the hospital and the general practitioners.

The cohort of 881 patients was divided into two age groups: 75 years and younger (low age group), and older than 75 years (high age group). Updated means of total cholesterol, LDL-cholesterol, HDL-cholesterol, triglycerides, and the cholesterol-HDL ratio were calculated for each individual from baseline to the end of follow-up by averaging the baseline values with the mean annual values. This technique is similar to the one used in the United Kingdom Prospective Diabetes Study (UKPDS) [17]. For example, at one year the updated mean total cholesterol is the average of the baseline and one year values and at three years it is the average of baseline, one year, two year and three year values. Eleven baseline variables were selected as possible confounders in the relationship between the lipid profile indices and mortality: gender, smoking (yes or no), body mass index (BMI), duration of diabetes, serum creatinine level, macrovascular complications (yes or no), albuminuria (yes or no), systolic blood pressure, use of lipid-lowering drugs (yes or no), use of antihypertensive drugs (yes or no), and age. Patients were considered to have macrovascular complications when they had a history of angina pectoris, myocardial infarction, percutaneous transluminal coronary angioplasty, coronary artery bypass grafting, stroke or transient ischaemic attack.

Continuous variables are represented as mean (± standard deviation) for normally distributed values and as median (interquartile range) for non-normally distributed values. Normality was evaluated using Q-Q plots. Nominal variables are represented as the total number of patients (percentage). A Cox proportional hazard model was used to investigate the relationship between the updated means of the lipid profile indices, as time dependent covariates, and mortality with and without adjustment for the selected confounders. The model was used in both age groups. The assumption of proportional hazards was checked by inspecting the Schoenfeld residual plots for each predictor variable. No substantial deviations from the plots were observed. Low cholesterol levels seen in patients close to death can influence the relationship between the lipid profile and mortality. Therefore, we performed additional analyses in which we excluded the deaths occurring in the first 4 years of follow-up. Analyses were repeated in strata according to diabetes duration. The diabetes duration variable was categorised into two different groups: diabetes of short duration (below the median value) and diabetes of long duration (the median value and higher). All analyses were performed with SPSS version 15.0.1 (SPSS inc., Chicago, Illinois, USA).

### Ethics Statement

The ZODIAC study and the informed consent procedure was approved by the local medical ethics committee of the Isala Clinics, Zwolle, The Netherlands. Verbal informed consent was obtained for all patients by the participating diabetes specialist nurses and the consent was documented in the patients records. According to Dutch law, written informed consent was not necessary for this type of study in 1998. All data were analyzed anonymously.

## Results

The baseline characteristics of the study population are presented in table 1. Diastolic blood pressure, BMI, total cholesterol, level of triglycerides and the proportion of smoking patients were lower in the high age group, whilst HDL-cholesterol and serum creatinine were higher. Also, diabetes duration was longer and complications were more prevalent. There were no differences between groups regarding glycemic control and the proportion of patients on oral glucose lowering agents and insulin treatment. In the entire cohort, 95 patients (11%) received LLT at the beginning of the study. During a median follow-up time of 9.8 years, LLT was started in 171 patients (31%) in the low age group, and in 26 patients (8%) in the high age group. Two hundred sixty-seven out of 326 patients (82%) died in the high age group and 198 out of 555 patients (36%) in the low age group. The proportions of cardiovascular mortality in the high and low age groups were 43% (114 out of 267) and 42% (83 out of 198), respectively.

**Table 1:** Baseline characteristics.

	60–75 years	>75 years	p-value
	*n = 555*	*n = 326*	
Age (years)	69 (65–72)	80 (77–83)	–
Male sex	230 (41)	118 (36)	0.124
Duration of T2DM (years)	6 (3–12)	8 (4–13)	0.001
Body mass index (kg/m^2^)	29.0 (±4.6)	27.7 (±4.4)	<0.001
Systolic blood pressure (mm Hg)	158.2 (±25.1)	156.0 (±24.0)	0.206
Diastolic blood pressure (mm Hg)	84.8 (±11.2)	81.5 (±10.8)	<0.001
Pulse pressure (mm Hg)	73.5 (±20.2)	74.6 (±18.9)	0.424
Total cholesterol (mmol/L)	5.7 (±1.1)	5.5 (±1.2)	0.009
Cholesterol-HDL ratio	5.3 (±1.5)	4.9 (±1.6)	0.001
LDL-cholesterol (mmol/L)	3.4 (±1.0)	3.3 (±1.0)	0.191
HDL-cholesterol (mmol/L)	1.1 (±0.3)	1.2 (±0.4)	0.016
Triglycerides (mmol/L)	2.6 (±1.5)	2.2 (±1.4)	<0.001
HbA1c (%)	7.5 (±1.4)	7.4 (±1.2)	0.205
Smoking	85 (15)	31 (10)	0.014
Albuminuria present	245 (44)	181 (56)	0.002
Macrovascular complications present	208 (37)	149 (46)	0.016
Receiving antihypertensive treatment	277 (50)	197 (60)	0.002
Receiving lipid-lowering treatment	80 (14)	15 (5)	<0.001
Receiving oral glucose lowering agents	406 (73)	234 (72)	0.659
Receiving insulin treatment	94 (17)	66 (20)	0.219
Serum creatinine (umol/L)	92 (82–104)	98 (86–117)	<0.001

Data are means (± SD), medians (interquartile range) or *n* (%). Chi-squared tests were used for categorical variables and independent t-tests or Mann-Whitney U tests, where appropriate, for continuous variables.

### Low Age Group

**Figure 2:**
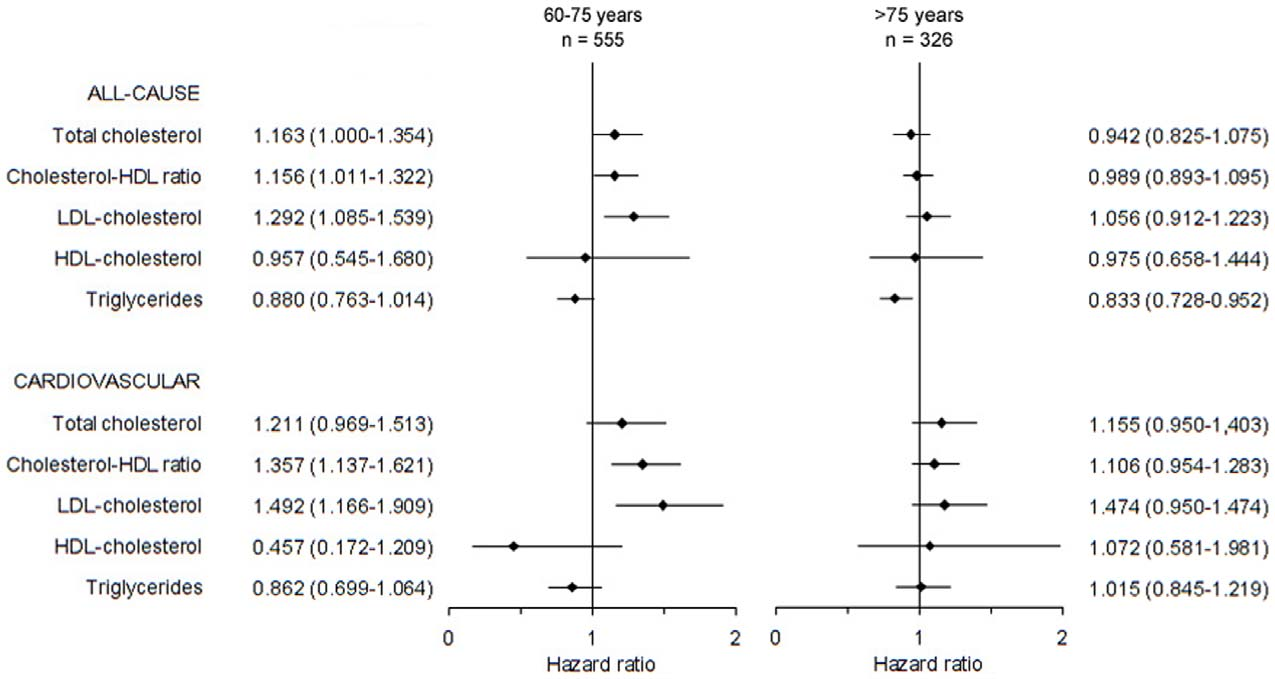
Combined forest plot: lipid profile and mortality. Hazard ratios and the 95% confidence intervals of the various lipid profile indices for all-cause and cardiovascular mortality in both age groups, adjusted for the selected confounders.

Figure 2 presents the hazard ratios, adjusted for the selected confounders, for all-cause and cardiovascular mortality in both age groups. An increase of the cholesterol-HDL ratio, as well as increased levels of LDL-cholesterol were associated with both increased all-cause and cardiovascular mortality in the low age group. An increase of 1 mmol/L in the level of LDL-cholesterol led to an increase of the hazard of cardiovascular mortality by 49% (95% confidence interval (CI) 17% to 91%). There was a non-significant positive relationship between total cholesterol and all-cause as well as cardiovascular mortality. The relationships for both HDL-cholesterol and triglycerides with mortality were also non-significant.

### High Age Group

Except for the triglyceride level, none of the other lipid profile indices were related to all-cause mortality in the high age group. The mortality risk decreased by 17% (95%CI 5% to 27%) for each 1 mmol/L higher level of triglycerides. All associations between the lipid profile indices and cardiovascular mortality were non-significant.

### Exclusion of Early Mortality

**Figure 3:**
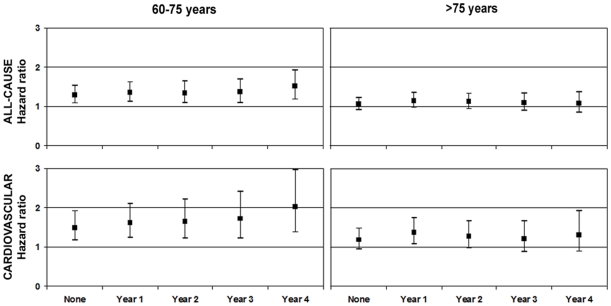
Exclusion of early mortality. Hazard ratios and their 95% confidence intervals (adjusted for confounders) of LDL-cholesterol for all-cause and cardiovascular mortality after exclusion of the deaths in the first follow-up years. Hazard ratios of several analyses in both age groups are presented: an analysis including all follow-up years, an analysis in which the first year of follow-up was excluded, an analysis in which the first two years of follow-up was excluded and so forth.

After exclusion of the deaths in the first years of follow-up the relationship between LDL-cholesterol and mortality became more pronounced in the low age group, but did not relevantly change in the group of the elderly diabetic patients (figure 3). The same effect was also seen regarding total cholesterol (data not shown). All other associations, including the one between triglycerides and all-cause mortality, did not relevantly change after exclusion of early mortality.

### Diabetes Duration

**Figure 4:**
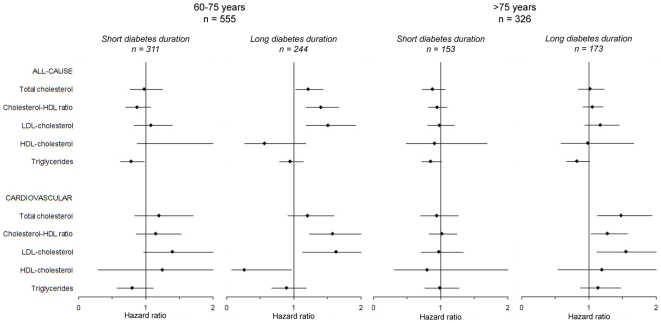
Combined forest plot: short versus long diabetes duration. Hazard ratios and the 95% confidence intervals of the various lipid profile indices stratified for diabetes duration (< median value versus median value and higher), adjusted for the selected confounders.

When the analyses were repeated in strata according to diabetes duration, the results differed from the overall results (figure 4). For patients in the low age group with diabetes of long duration (≥6 years), the cholesterol-HDL ratio and LDL-cholesterol were positively associated with both all-cause and cardiovascular mortality. HDL-cholesterol was inversely related to cardiovascular mortality in this group. Only the triglyceride level was related to all-cause mortality for patients in the low age group with diabetes of short duration: this relationship was inverse.

The lipid profile was not predictive of all-cause mortality in the high age group, regardless of diabetes duration. An increase of the cholesterol-HDL ratio, as well as increased levels of total and LDL-cholesterol were associated with increased cardiovascular mortality in elderly patients with diabetes of long duration (≥8 years).

## Discussion

After a median follow-up time of almost 10 years, the overall results showed that higher lipid levels were unrelated to increased all-cause and cardiovascular mortality in patients with T2DM aged over 75 years. However, the results were different when stratified for diabetes duration. In elderly patients with a diabetes duration of 8 years or longer, higher lipids were related to increased cardiovascular mortality. The overall results showed an inverse relationship between the triglyceride level and all-cause mortality in the high age group. An increase of 1 mmol/L led to a decrease in mortality risk of 17%. In younger patients we found relations as one would expect, e.g. a higher LDL-cholesterol is related to increased mortality.

With increasing age, age itself becomes more important in the prediction of mortality. As a consequence, the predictive value of other risk factors like lipid profile abnormalities becomes smaller. Interestingly, in the high age group we only found positive relationships in patients with diabetes of long duration. These results correspond with a previous study that showed that patients with middle age- and elderly onset diabetes appear to represent distinct groups with differing burdens of disease [18]. One could hypothesize that patients with diabetes of long duration may represent a selected group with a worse cardiovascular risk profile compared to patients with diabetes of short duration. So, the predictive value of the lipid profile may be higher in patients with diabetes of long duration, because the total cardiovascular mortality risk in this group may be higher. However, this hypothesis could not be confirmed in our cohort: the proportions of cardiovascular mortality for the elderly patients with diabetes of short and long duration were 47% and 54%, respectively (p = 0.907).

Low total cholesterol has been described before as a marker of increased mortality in the general elderly population [10]–[14]. We found an inverse relationship between the triglyceride level and all-cause mortality in both the total group of patients older than 75 years, and in a subgroup of younger patients with diabetes of short duration. Various explanations have been proposed for the absence of a positive relationship and sometimes an inverse relationship, between lipids and mortality. Lower cholesterol levels may represent occult disease or may be a signal of declining health [13]. It is also possible that subjects surviving long enough with a higher cholesterol are less susceptible to the adverse consequences of dyslipidemia; those individuals susceptible to the effects of high cholesterol will probably die before reaching the age of 75 years. Those individuals who survive, represent a selected group with lower cholesterol levels and with a genetic makeup or other factors protecting them from the effects of higher cholesterol levels [12]. To adjust for the possible confounding effects of co-morbidity and frailty, we performed additional analyses in which we excluded deaths occurring in the first years of follow-up. The relationship between the triglyceride level and all-cause mortality remained inverse. All other lipid profile indices remained non-predictive of mortality in the high age group.

In very old people from the general population with no history of cardiovascular disease, classic risk factors included in the Framingham risk score did not predict cardiovascular mortality [19]. This study and the previously mentioned studies, that showed an inverse relationship between cholesterol and mortality, question the ability of lipid profile abnormalities to identify elderly patients at high risk of (cardiovascular) mortality [10]–[14], [19]. It seems therefore that classic risk factors may have different consequences when assessed in elderly patients. However, it is important to emphasize that older adults exhibit widely heterogeneous health status, ranging from healthy to frail [20], [21]. In a guideline for improving diabetes care in older adults, it has been suggested to treat lipid profile abnormalities only after overall health status has been considered [20]. Furthermore, it was stated that only 2 to 3 years are required to see benefits from LLT [20]. Physicians caring for elderly diabetic patients should take co-morbidity and the estimated life expectancy into account when setting treatment goals for the individual patient. In our opinion, it is plausible that there are specific groups in which lipids are predictive of mortality and LLT is beneficial; perhaps in elderly patients with diabetes of long duration.

The proportion of patients receiving LLT at the start of the ZODIAC-study was small in both age categories. As a consequence, it was not possible to perform separate analyses for the effects of LLT on mortality. One has to remember that this study started in 1998, and the treatment goals for the lipid profile in patients with T2DM are more stringent nowadays. Whether LLT is beneficial in elderly patients with T2DM remains unanswered in this study. Several trials have investigated the effects of statins in patients with T2DM. One large randomized controlled trial, including 5963 diabetic patients, showed beneficial effect of LLT on major vascular events in elderly T2DM patients (≥65 years) [22]. Furthermore, a meta-analysis investigating the effects of LLT in diabetic patients also performed a separate analysis for patients aged 65 years and older [4]. The relative risk for major vascular events was 0.81 (95%CI 0.71 to 0.92) in favor of LLT. Again, only a few studies had included patients aged over 75 years.

The main limitation of this study is its observational design, which prevents us drawing conclusions about causality. Another limitation is that we are not informed about the proportion of non-fasting lipid profiles during the yearly assessments in our study population. However, a fasting blood sample is not necessary for a reliable measurement of total cholesterol and HDL-cholesterol.

Our study also has notable strengths. Firstly, we used the various lipid profile indices as time dependent covariates and by means of this analysis we corrected for changes over time. Secondly, with a median follow-up of almost 10 years, we were able to observe a substantial number of deaths to base our conclusions on.

To our knowledge this is the first prospective observational study which specifically investigated the relationship between lipids and mortality in a cohort of T2DM patients older than 75 years. Although the lipid profile was not predictive in the overall group of elderly patients, higher lipids were related to increased cardiovascular mortality in patients with diabetes of long duration. So, it may be necessary to formulate different treatment goals for different patients groups. In order to identify these specific groups and to make valid recommendations concerning LLT, a randomized controlled trial or a meta-analysis concerning this specific population is mandatory.
